# Engineered Curli Nanofilaments as a Self‐Adjuvanted Antigen Delivery Platform

**DOI:** 10.1002/adhm.202300224

**Published:** 2023-04-29

**Authors:** Félix Lamontagne, Dominic Arpin, Mélanie Côté‐Cyr, Vinay Khatri, Philippe St‐Louis, Laurie Gauthier, Denis Archambault, Steve Bourgault

**Affiliations:** ^1^ Department of Chemistry Université du Québec à Montréal C.P.8888, Succursale Centre‐Ville Montreal H3C 3P8 Canada; ^2^ Quebec Network for Research on Protein Function Engineering and Applications (PROTEO) Quebec H3C 3P8 Canada; ^3^ The Swine and Poultry Infectious Diseases Research Centre (CRIPA) Saint‐Hyacinthe J2S 2M2 Canada; ^4^ The Center of Excellence in Research on Orphan Diseases – Fondation Courtois (CERMO‐FC) Montreal H3C 3P8 Canada; ^5^ Department of Biological Sciences Université du Québec à Montréal C.P.8888, Succursale Centre‐Ville Montreal H3C 3P8 Canada

**Keywords:** antigen delivery platforms, curli‐specific gene A, immunostimulants, influenza A virus, nanofilaments, nanovaccines

## Abstract

Proteinaceous nanoparticles constitute efficient antigen delivery systems in vaccine formulations due to their size and repetitive nature that mimic most invading pathogens and promote immune activation. Nonetheless, the coadministration of an adjuvant with subunit nanovaccines is usually required to induce a robust, long‐lasting, and protective immune response. Herein, the protein Curli‐specific gene A (CsgA), which is known to self‐assemble into nanofilaments contributing to bacterial biofilm, is exploited to engineer an intrinsically immunostimulatory antigen delivery platform. Three repeats of the M2e antigenic sequence from the influenza A virus matrix 2 protein are merged to the N‐terminal domain of engineered CsgA proteins. These chimeric 3M2e‐CsgA spontaneously self‐assemble into antigen‐displaying cross‐*β*‐sheet nanofilaments that activate the heterodimeric toll‐like receptors 2 and 1. The resulting nanofilaments are avidly internalized by antigen‐presenting cells and stimulate the maturation of dendritic cells. Without the need of any additional adjuvants, both assemblies show robust humoral and cellular immune responses, which translate into complete protection against a lethal experimental infection with the H1N1 influenza virus. Notably, these CsgA‐based nanovaccines induce neither overt systemic inflammation, nor reactogenicity, upon mice inoculation. These results highlight the potential of engineered CsgA nanostructures as self‐adjuvanted, safe, and versatile antigen delivery systems to fight infectious diseases.

## Graphical abstract

The protein Curli‐specific gene A, which is known to self‐assemble into nanofilaments contributing to bacterial biofilm, is engineered to design an intrinsically immunostimulatory antigen delivery nanoplatform for vaccination purpose. These assemblies harboring a conserved influenza A antigen show robust humoral and cellular immune responses, which translate into complete protection against a lethal experimental infection with the H1N1 influenza virus.

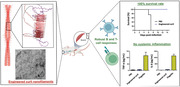

## 1 Introduction

Vaccination has been one of the most significant medical advances for human health and has efficiently alleviated the economic losses associated with infectious diseases afflicting domesticated animals.^[^ ^1^ ^]^ To address potential safety concerns associated with the usage of live‐attenuated and inactivated pathogens, vaccine technologies inducing a robust and effective immune response while being safe have emerged.^[^ ^2^ ^]^ Among them, subunit vaccines, which are composed of defined microbial antigens, have shown to be effective at inducing a protective immune response combined with a high safety profile. Purified antigens are usually weakly immunogenic, requiring the usage of immunostimulatory agents, known as adjuvants, and delivery systems to generate robust antigen‐specific responses.^[^ ^3^ ^]^ Particularly, the conjugation of an antigen to a nanoparticle is an efficient strategy to boost its immunogenicity by increasing uptake by antigen‐presenting cells (APCs), improving stability and/or enhancing retention at the draining lymph nodes.^[^ ^3^, ^4^ ^]^ Moreover, the organized multivalent display of the antigen on the nanoparticle can trigger the cross‐linkage of B cell receptors (BCRs), which enhances B cell activation and antibody production.^[^ ^5^ ^]^ Owing to their size, nanoparticles can diffuse passively in the lymphoid system while not distributing into blood capillaries, ultimately limiting potential systemic toxicity observed with low molecular weight immunomodulators.^[^ ^6^ ^]^ A diversity of materials has been evaluated as antigen‐delivery nanosystems, such as inorganic particles, polymers, liposomes, and proteinaceous assemblies.^[^ ^7^ ^]^ In contrast to inorganic and polymeric particles, protein nanoparticles are attractive antigen carriers due to their biocompatibility, biodegradability, biological stability, and the repetitive nature of the assembly.^[^ ^8^ ^]^ Protein‐based nanovaccines include virus‐like particles^[^ ^9^ ^]^ as well as bacterial self‐assembling proteins, such as ferritin^[^ ^10^ ^]^ and lumazine synthase.^[^ ^11^ ^]^ However, these proteinaceous delivery nanosystems usually have no intrinsic immunostimulatory activity, requiring the use of an adjuvant in the vaccine formulations.^[^ ^5^, ^10^ ^]^ Thus, there is still a critical need to identify novel protein‐based nanoparticles with intrinsic immunostimulatory properties for efficient antigen delivery.

The protein Curli‐specific gene A (CsgA), which is expressed by numerous enteric bacteria,^[^ ^12^ ^]^ constitutes the main extracellular matrix component contributing to biofilm formation.^[^ ^13^ ^]^ Secretion and CsgB‐templated self‐assembly of CsgA into nanofilaments on the outer membrane provide structural support for the colonization of tissues and of inert surfaces.^[^ ^14^ ^]^ CsgA is composed of five imperfect repeating units, termed R1 to R5, that fold into a *β*‐sheet‐turn‐*β*‐sheet motif and stack on top of one another, with the resulting *β*‐helix supramolecular structure being stabilized by extensive intermolecular hydrogen bond ladders involving Asn and Gln amide side chains.^[^ ^15^ ^]^ This cross‐*β*‐sheet quaternary conformation, often associated with amyloid fibrils, confers robust physical properties and high mechanical resistance to proteolysis.^[^ ^16^ ^]^ In vitro, recombinant CsgA monomers spontaneously self‐assemble into long and unbranched fibrils with similar (supra)structural architecture to their counterparts assembled at the bacterial membrane.^[^ ^17^ ^]^ CsgA has shown robust self‐assembly capacity under a variety of conditions^[^ ^18^ ^]^ and upon conjugation with large proteins,^[^ ^19^ ^]^ indicating that high molecular weight antigens could be conjugated to CsgA, while maintaining its ability to form cross‐*β* filaments. Interestingly, bacterial curli fibrils are recognized by the innate immune system, leading to the expression and secretion of chemokines and cytokines.^[^ ^20^ ^]^ Furthermore, it was reported that CsgA assemblies can activate the membrane‐bound Toll‐like receptor 2 (TLR2) and the cytosolic inflammasome Nod‐like receptor pyrin 3 (NLRP3), inducing the activation and maturation of APCs and the release of IL‐1*β* and IL‐6.^[^ ^21^ ^]^ Recent studies have exposed that the cross‐*β*‐sheet quaternary motif constitutes a conformational agonist of heterodimeric TLR2‐TLR1 and TLR2‐TLR6.^[^ ^22^ ^]^ Whereas the potency of CsgA to activate innate immunity, a prerequisite for robust adaptive immune responses, has been reported, the usage of CsgA filaments as a biocompatible, biologically stable, versatile, and robust self‐adjuvanted antigen delivery nanosystem has never been investigated.

In this context, the present study aims at evaluating the use of CsgA‐based filaments for the preparation of intrinsically adjuvanted subunit nanovaccines. In contrast to conventional nanoparticles, CsgA assemblies would function as both an immunopotentiator and a delivery system with a repetitive antigen display on the surface. To assess this hypothesis, three repeats of the ectodomain of the matrix 2 protein (M2e) of the influenza A virus (IAV) were merged to the N‐terminal domain of full‐length and truncated CsgA proteins, and the resulting chimeric nanoassemblies were evaluated as vaccine formulations against an IAV experimental infection. Conjugation of the antigen did not affect the capacity of CsgA‐derived monomers to self‐assemble into nanofilaments that engage the heterodimeric TLR2‐TLR1 and activate APCs. Mice immunized with CsgA‐based vaccines showed robust immune responses against the grafted M2e antigen, leading to protection against H1N1 IAV infection. Additionally, CsgA‐based formulations did not lead to overactivation of the inflammatory response and exhibited no adverse effects upon immunization. These results indicate that CsgA‐engineered nanofilaments constitute a novel, effective, safe, and self‐adjuvanted vaccine platform for the delivery of antigenic determinants.

## 2 Results and Discussion

### 2.1 Design and Characterization of CsgA‐Based Nanofilaments

**Figure 1:**
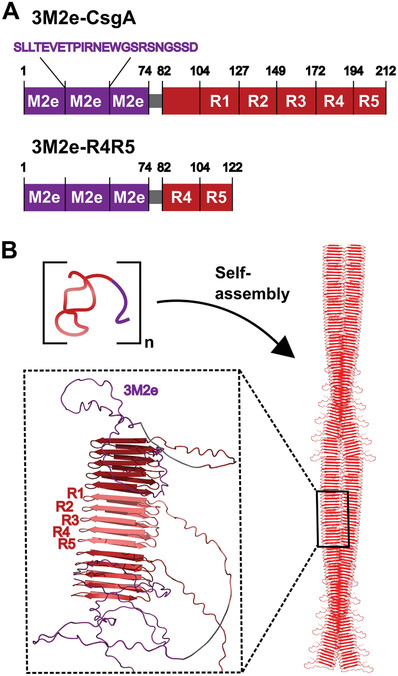
A) Schematic representations of the primary structure of CsgA and the fragment R4R5 in fusion with three tandem repeats of the M2e antigenic sequence from IAV. B) Self‐assembly of the chimeric CsgA‐based proteins into cross‐*β*‐sheet fibrils. The structural organization of the assemblies was predicted using ColabFold:AlphaFold2‐multimer.^[^ ^28^ ^]^

The ectodomain of the IAV M2 protein, M2e, is a potential linear epitope‐containing peptide candidate for an universal flu vaccine, owing to its highly conserved sequence amongst various virus subtypes.^[^ ^23^ ^]^ Several studies have revealed that a vaccination strategy based on the M2e antigenic sequence induced cross‐protection against various subtypes of influenza A virus.^[^ ^24^ ^]^ Although it is expressed at the surface of the virions, the M2e antigen is not neutralizing per se, but it is targeted by antibodies at the surface of IAV‐infected cells and promotes their elimination via antibody‐dependent cellular phagocytosis by alveolar macrophages^[^ ^25^ ^]^ and antibody‐dependent cellular cytotoxicity by natural killer cells.^[^ ^26^ ^]^ However, the M2e peptide is poorly immunogenic, and adjuvanted delivery strategies are needed to enhance its immunogenicity. In the present study, three repetitions of the M2e sequence from IAV (strain A/Puerto Rico/8/1934 H1N1) were fused to the N‐terminal domain of CsgA‐derived proteins, and flexible linkers (GGGSGGGS) were added between the 3M2e and the self‐assembling units (**Figure** **1**). To prevent any spontaneous disulfide bond formation involving M2e, the two Cys residues of the M2e sequence were mutated to Ser (SLLTEVETPIRNEWGSRSNGSSD), a modification known not to impact the immune recognition.^[^ ^27^ ^]^ Moreover, to address the challenges and potential issues with the expression and handling of full‐length CsgA, including the formation of large amorphous aggregates that tend to precipitate out of solution and stick to surfaces, a truncated version of CsgA containing only the fourth and fifth repeating units (R4R5) was engineered. The R4R5 units have been shown to be sufficient for immunostimulation,^[^ ^21d^ ^]^ while potentially mitigating CsgA aggregation due to the lack of the first and third repetitions (R1 & R3), which are highly amyloidogenic.^[^ ^17b^ ^]^ Both chimeric proteins were expressed in *Escherichia coli* with a HisTag at the C‐terminus and purified under denaturing conditions by immobilized metal affinity chromatography (IMAC) (Figure S1, Supporting Information), yielding proteins of 23 and 14 kDa for 3M2e‐CsgA and 3M2e‐R4R5, respectively (Figure S2, Supporting Information).

**Figure 2:**
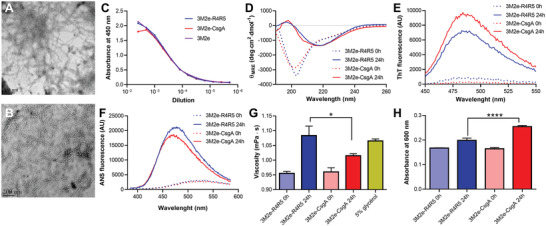
Self‐assembly of CsgA‐based chimeric proteins into cross‐*β*‐sheet nanofilaments. A,B) Negative stain electron microscopy of assembled (A) 3M2e‐CsgA and (B) 3M2e‐R4R5. The scale bar is 200 nm. C) Antigen accessibility on CsgA‐based assemblies by indirect ELISA with anti‐M2e antibody. D) Far‐UV CD spectra of freshly purified (0 h) and assembled (24 h) chimeric proteins. E,F) Fluorescence spectra of (E) ThT and (F) ANS after excitation at 440 and 370 nm, respectively. G) Viscosity and H) turbidity analysis of freshly purified (0 h) and assembled (24 h) 3M2e‐CsgA and 3M2e‐R4R5. *n* = 3 per group, data show the means ± S.D. Statistical significance was established using a student's *t*‐test with *P < 0.05, ****P < 0.0001. (A–H) Proteins were incubated in sterile PBS for 24 h at RT at a concentration of 600 µg mL^−1^ under fully quiescent conditions.

After the elimination of chaotropic agents by size exclusion chromatography, freshly purified 3M2e‐CsgA and 3M2e‐R4R5 were incubated at room temperature (RT) under fully quiescent conditions at a concentration of 600 *µ*g mL^−1^ in sterile PBS to initiate self‐assembly. Negative stain transmission electron microscopy (TEM) showed that after 24 h incubation, both proteins assembled into linear filaments of up to a few micrometers in length and diameter ranging from 5 to 10 nm (**Figure** **2**A,B), with morphology similar to what has been previously reported for CsgA assemblies.^[^ ^17b^ ^]^ Nonetheless, it is worth mentioning that large clumps of nanofilaments, which tend to precipitate, were observed by atomic force microscopy (AFM) and TEM for the full‐length 3M2e‐CsgA preparations (Figures S3 and S4, Supporting Information). These large aggregates were absent for the 3M2e‐R4R5 formulation (Figures S3 and S4, Supporting Information), in which nanofilaments remained dispersed and in suspension. Thus, the length distribution of assemblies could only be determined for the R4R5 preparations, revealing an average length of 307.9 ± 216.9 nm (Figure S5, Supporting Information). An anti‐M2e enzyme‐linked immunosorbent assay (ELISA) confirmed that the antigen was accessible to antibodies and maintained its antigenicity following assembly into filaments (Figure 2C). Circular dichroism spectroscopy revealed that 3M2e‐CsgA and 3M2e‐R4R5 were mostly unstructured immediately after their purification and shifted to a *β*‐sheet‐rich secondary conformation after 24 h incubation, as exemplified by the transition from spectra characterized with a single minimum at ≈200 nm to spectra with a minimum at 220 nm and a maximum at ≈198 nm, typical of cross‐*β* assemblies (Figure 2D). Fluorescence of thioflavin T (ThT), a small dye that binds selectively to cross‐*β*‐sheet quaternary motifs,^[^ ^29^ ^]^ further confirmed self‐assembly (Figure 2E). Moreover, the dye 8‐anilinonaphthalene1‐sulfonic acid (ANS), whose fluorescence quantum yield increases upon binding to protein hydrophobic domains, revealed the formation of accessible hydrophobic pockets upon fibril formation, which were absent in the monomeric/prefibrillar protein solutions (Figure 2F). The ANS fluorescence signal obtained for 3M2e‐R4R5 was stronger than that of 3M2e‐CsgA, which could be associated with lower degree of exposure of solvent‐accessible hydrophobic clusters in 3M2e‐CsgA preparations, as these assemblies tend to form dense networks of clumped filaments, as observed by TEM and AFM. Protein self‐recognition and aggregation are known to increase solution viscosity, a property that can be beneficial for vaccine formulations by enhancing retention time at the injection site and on mucosal surface.^[^ ^30^ ^]^ Accordingly, we analyzed the viscosity of 3M2e‐CsgA and 3M2e‐R4R5 solutions immediately after purification (0 h), perhaps under monomeric form, and after 24 h incubation at RT. Both protein solutions showed an increase in viscosity associated with the self‐assembly process, with the 3M2e‐R4R5 solution being significantly more viscous compared to 3M2e‐CsgA (Figure 2G). In fact, the solution of 3M2e‐R4R5 filaments at a concentration of 600 µg mL^−1^ had comparable viscosity to a 5% glycerol solution and tube inversion further exposed the high viscosity of the 3M2e‐R4R5 formulations (Figure S6, Supporting Information). Finally, the colloidal properties of protein assemblies are known to increase the scattering of visible light, and turbidity is often used to follow protein self‐assembly. At a concentration of 600 µg mL^−1^, an increase of absorbance at 600 nm was observed after self‐assembly, with the 3M2e‐CsgA solution being significantly more turbid compared to 3M2e‐R4R5 (Figure 2H). Overall, these results indicate that the self‐assembly of CsgA and its truncated R4R5 fragment tolerates the addition of an antigen at their N‐terminus and that the M2e epitopes are accessible at the filament surface. Particularly, deleting the R1 to R3 segments of CsgA (R4R5), led to a suspension of well‐defined nanofilaments that increase solution viscosity, a property that could potentially improve the efficacy of the vaccine formulation.

### 2.2 CsgA Nanofilaments Engage the Heterodimer TLR2‐TLR1 and Promote IL‐1*β* Secretion

**Figure 3:**
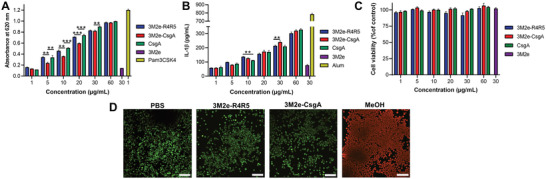
CsgA‐based assemblies activate TLR2 and induce IL‐1*β* secretion independently of cell death. A) TLR2‐TLR1 stimulation by CsgA assemblies. HEK‐Blue cells expressing the heterodimer TLR2‐TLR1 were exposed to nanofilaments for 16 h and activation was measured using SEAP reporter. B) J774.A1 murine macrophages were incubated for 16 h with nanofilaments and levels of IL‐1*β* in the supernatant were measured by ELISA. C,D) Viability of J774.A1 macrophages upon treatment with CsgA‐based nanofilaments. (C) Cells were treated with nanofilaments for 16 h and metabolic activity was measured by resazurin reduction. (D) Representative fluorescence microscopy images showing the distribution of live (green) and dead (red) J774.A1 cells after treatment with 30 µg mL^−1^ of nanofilaments for 16 h. Scale bar: 100 µm. (A–C) *n* = 3 to 5 per group, data represent the mean ± S.D. and statistical significance was analyzed with a one‐way ANOVA with Tukey's multiple comparisons tests (**P < 0.01; ***P < 0.001).

TLR‐signaling is involved in the activation and maturation of immune cells and in the induction of a robust antigen‐specific immune response.^[^ ^31^ ^]^ TLR2, a cell surface receptor that forms heterodimers with TLR1 or TLR6, is widely expressed on endothelial cells and APCs.^[^ ^31^ ^]^ The TLR2 recognizes a diversity of ligands, including lipoproteins, peptidoglycans, porins, and the cross‐*β*‐sheet quaternary motif of protein assemblies.^[^ ^22^, ^32^ ^]^ Supplementing vaccine formulations with TLR2 agonists, such as Pam2CSK4^[^ ^33^ ^]^ and Pam3CSK4,^[^ ^34^ ^]^ have been shown to promote the recruitment of immune cells and the maturation of APCs, ultimately inducing a robust cellular and humoral immune response. CsgA fibrils are known to engage the heterodimer TLR2‐TLR1 in bone‐marrow‐derived macrophages, and the C‐terminal R4R5 region is sufficient for its activation.^[^ ^21b,d^ ^]^ To evaluate that the addition of the 3M2e antigenic motif did not impede TLR2 activation, the HEK‐Blue hTLR2‐TLR1 reporter cell line, which expresses NF‐*κ*B/AP‐1‐inducible secreted embryonic alkaline phosphatase (SEAP) reporter, was used. 3M2e‐CsgA and 3M2e‐R4R5 nanofilaments showed comparable concentration‐dependent activation of the TLR2‐TLR1‐mediated NF‐*κ*B signaling, which was equivalent to the response obtained with unmodified CsgA filaments (**Figure** **3**A). This result indicates that the addition of an antigen at the surface of the CsgA filaments does not affect its previously demonstrated TLR2‐agonist properties. Following binding and activation of the TLR2‐TLR1, the ligand and heterodimeric receptor are conjointly endocytosed, and the cross‐*β*‐sheet fibrils can leak out of endosomes into the cytosol and activate the NLRP3 inflammasome, inducing the production of pro‐IL‐1*β* and subsequent cleavage into IL‐1*β*.^[^ ^21a^ ^]^ To assess IL‐1*β* secretion associated with potential inflammasome activation, J774.A1 murine macrophages^[^ ^35^ ^]^ were exposed to increasing concentration of fibrils and the level of IL‐1*β* in the media after 16 h exposure was measured by ELISA. CsgA nanoassemblies induced equivalent concentration‐dependent production of IL‐1*β*, suggesting that the inflammasome activating properties were retained upon N‐terminal conjugation of antigenic determinants (Figure 3B). Considering that IL‐1*β* secretion can be associated with pyroptosis^[^ ^36^ ^]^ and that some amyloid fibrils can be cytotoxic,^[^ ^37^ ^]^ we probed the viability of J774.A1 macrophages after 24 h exposition to CsgA filaments by measuring the metabolic activity and by means of the LIVE/DEAD assay. Resazurin reduction revealed that CsgA‐based filaments were fully cytocompatible, with no decrease of cellular viability observed at the highest concentration evaluated, i.e., 60 *µ*g mL^−1^ (Figure 3C). Additionally, as observed by fluorescence microscopy following the LIVE/DEAD assay, 24 h treatment with 30 µg mL^−1^ of 3M2e‐CsgA, or 3M2e‐R4R5, filaments did not increase the number of red cells, nor decrease the number of green cells, suggestive of absence of cytotoxicity (Figure 3D). Green fluorescence of calcein‐AM is associated with intracellular esterase activity, while red fluorescence of ethidium homodimer‐III is linked to the loss of plasma membrane integrity. This observation corroborates with the well‐known cytocompatibility of stable and organized cross‐*β*‐sheet proteinaceous nanostructures.^[^ ^38^ ^]^ Taken together, these results indicate that 3M2e‐CsgA and 3M2e‐R4R5 nanofilaments show robust capacity to stimulate key players of innate immunity, without noticeable cytotoxicity.

### 2.3 CsgA‐Based Nanofilaments are Efficiently Uptaken by APCs and Induce Maturation of Dendritic Cells

**Figure 4:**
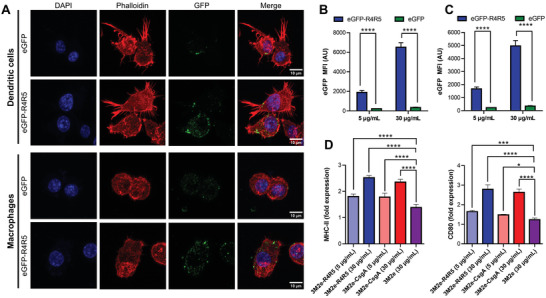
Cellular uptake of CsgA nanofilaments by APCs and maturation of DCs. Internalization by A,B) DC2.4 dendritic cells and A,C) J774.A1 macrophages analyzed by (A) confocal microscopy and (B,C) flow cytometry. (A–C) Cells were treated with 5 (B,C) and/or 30 µg mL^−1^ (A–C) for 3 h with eGFP‐R4R5 nanofilaments or eGFP followed by extensive washing. For flow cytometry, trypan blue was added immediately before analysis. D) Flow cytometry analysis of DC2.4 cells following treatment with CsgA‐based assemblies for 16 h. Fold expression is determined relative to the PBS vehicle control. (B‐D) *n* = 3 to 5 per group, data represent the mean ± S.D., and statistical significance was analyzed with a one‐way ANOVA with Tukey's multiple comparisons tests (*P < 0.05; ***P < 0.001; ****P < 0.0001).

The cellular uptake of antigens by APCs is an essential step for the presentation of antigen‐derived peptides on major histocompatibility complex (MHC) and the induction of an antigen‐specific adaptive immune response. In comparison to their soluble counterpart, the conjugation of an antigen to a nanoparticle generally increases its internalization by APCs,^[^ ^39^ ^]^ an effect often associated with the increase of molecular size and/or the repetitive nature of the assemblies.^[^ ^4^, ^40^ ^]^ To observe the internalization of CsgA‐based filaments, the 3M2e antigen was replaced with the enhanced green fluorescent protein (eGFP), and the resulting fluorescent assemblies were used for confocal fluorescence microscopy and flow cytometry analysis. Bearing in mind that the eGFP in fusion with full‐length CsgA, or R4R5, did not refold properly following treatment with the chaotropic agent needed for purification (data not shown), the eGFP‐CsgA and eGFP‐R4R5 chimeric proteins were purified under native conditions. However, full‐length CsgA conjugated to eGFP readily aggregated into large, insoluble, and amorphous aggregates in the purification process, precluding analysis with defined fibrils. Accordingly, only the eGFP‐R4R5 chimeric protein could be used to evaluate the internalization of CsgA‐based nanofilaments by APCs. The self‐assembly of the R4R5 fragment tolerated the addition of a large protein as eGFP (Figures S7 and S8, Supporting Information), highlighting the robust self‐recognition properties of the truncated protein and indicating that large conformational antigens can be displayed on R4R5 filaments. Moreover, the eGFP‐R4R5 filaments activated the TLR2‐TLR1 in a concentration‐dependent manner (Figure S9, Supporting Information), suggesting that the eGFP‐R4R5 assemblies retain the immunostimulating properties associated with the cross‐*β* quaternary organization. DC2.4 dentritic cells and J774.A1 macrophages were respectively incubated for 3 h with 5 and 30 *µ*g mL^−1^ of eGFP‐R4R5 filaments and soluble eGFP before analysis by confocal fluorescence microscopy and flow cytometry. As observed by confocal microscopy conjugation of eGFP to R4R5 filaments drastically increased its internalization by both macrophages and dendritic cells, with the soluble eGFP being not discernible in APCs (**Figure** **4**A; Figures S10 and S11, Supporting Information). Moreover, flow cytometry analyses further exposed that the eGFP‐R4R5 filaments were internalized by both macrophages and DCs, whereas the soluble eGFP was poorly uptaken (Figure 4B,C; Figures S12 and S13, Supporting Information). Trypan blue was added to the flow cytometry buffer to quench any extracellular fluorescence and to differentiate internalized assemblies from membrane‐bound filaments, validating that the fluorescence signal was intracellular. Following antigen uptake and processing, DCs undergo maturation and upregulate the expression of MHC and costimulatory molecules.^[^ ^41^ ^]^ Accordingly, DC2.4 cells were respectively treated with 3M2e‐CsgA and 3M2e‐R4R5 filaments for 16 h, and the expression of MHC‐II and of the costimulatory molecule CD80 was evaluated by immunohistochemistry. Our results showed an increase of more than twofold for CD80 and MHC‐II expression upon 16 h treatment with both nanofilaments compared to the PBS vehicle control (Figure 4D). Taken together, these results show that CsgA‐based filaments are efficiently internalized by APCs and induce the maturation of DCs, which is a critical step for T cell activation and the induction of a robust antigen‐specific immune response. Nonetheless, it will now be important to validate the cellular uptake and the immunomodulation properties of these nanofilaments using isolated bone marrow derived DCs and macrophages.

### 2.4 3M2e‐R4R5 Nanofilaments Induce a Robust Anti‐M2e Specific Humoral Immune Response and Protect Mice against IAV Infection

**Figure 5:**
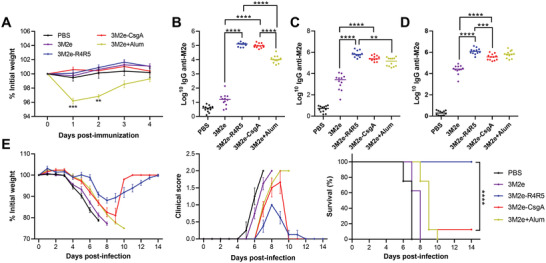
Intramuscular immunization with 3Me2‐R4R5 nanofilaments induces a robust anti‐M2e antibody response and protects mice against IAV infection. A–E) Mice were immunized intramuscularly with 18 µg of 3M2e (with or without 50% (v/v) Alum), 30 µg of 3M2e‐R4R5, or 50 µg of 3M2e‐CsgA. (A) Weight loss after primary immunization. (B–D) Total anti‐M2e IgG in mice sera 14 days after (B) primary immunization, (C) 1st boost, and (D) 2nd boost. (A–D) Statistical significance between groups was established using one‐way ANOVA with Tukey's multiple comparisons tests (**P < 0.01; ***P < 0.001; ****P < 0.0001). (E) Two weeks after the 3rd immunization, mice were inoculated intranasally with 5× LD_50_ of IAV H1N1. Mice were monitored daily to evaluate weight loss and clinical scores. *n* = 8 or 12 per group, data represent mean ± S.E.M. and statistical significance was obtained following a log‐rank Mentel‐Cox test (****P < 0.0001).

The immunostimulatory properties of CsgA‐based nanofilaments were evaluated in vivo by immunizing mice intramuscularly and measuring the M2e‐specific humoral immune response. Mice received three doses of equimolar concentration of 3M2e (18 µg of 3M2e; 30 µg of 3M2e‐R4R5; 50 µg of 3M2e‐CsgA) at 14‐days intervals. Sera were collected from the saphenous vein 13 days after each immunization and the anti‐M2e specific antibody response was measured by indirect ELISA. Following the first immunization, a significant decrease in weight was observed in mice that received the 3M2e protein supplemented with aluminum salts (Alum), which is commonly used as a vaccine adjuvant in human (**Figure** **5**A). In sharp contrast, mice that were immunized with CsgA‐based nanofilaments did not show any weight loss, an initial indication of innocuity. Following the first immunization, mice that received 3M2e‐CsgA and 3M2e‐R4R5 formulations had significantly higher level of anti‐M2e total IgG compared to mice that received soluble 3M2e in combination, or not, with Alum (Figure 5B). After the first boost, only mice that received the 3M2e‐R4R5 vaccine had significantly higher antibody titers than the 3M2e + Alum immunized mice (Figure 5C). Interestingly, following the third immunization, mice that received the 3M2e‐R4R5 formulation had significantly higher anti‐M2e IgG levels compared to mice immunized with the 3M2e‐CsgA vaccine (Figure 5D). According to the observed robust M2e‐specific antibody responses conferred by CsgA‐based nanovaccines, the capacity of the formulations to protect mice from an experimental IAV challenge was evaluated. Fourteen days after the last immunization, mice were infected by intranasal instillation with 5× the median lethal dose (LD_50_) of the influenza strain A/H1N1/Puerto Rico/8/1934. Weight loss and clinical signs of infection, including temperature, activity, and posture (Table S1, Supporting Information), were monitored daily, and mice that lost more than 20% of their weight were sacrificed and reported as dead animals. Eight to ten days postinfection, mice immunized with the PBS vehicle control and the 3M2e proteins, with or without Alum, showed 100% mortality (Figure 5E). The 3M2e‐CsgA formulation provided no significant protection, as only 12.5% of mice survived. In sharp contrast, immunization with 3M2e‐R4R5 conferred 100% survival with a mean weight loss of ≈10% and moderate clinical signs. 3M2e‐R4R5‐immunized mice regained their initial weight and showed no clinical signs 14 days after the infection. These results highlight the efficacy of the protection conferred by the 3M2e‐R4R5 nanovaccine against H1N1 IAV infection. It would be interesting to further evaluate if the coadministration of an adjuvant, such as Alum, with the immunostimulatory CsgA‐based platforms could act in synergy and potentiate the antigen‐specific immune response and the associated protection.

### 2.5 3M2e‐R4R5 Nanofilaments Induce a Balanced Th1/Th2 M2e‐Specific Immune Response

**Figure 6:**
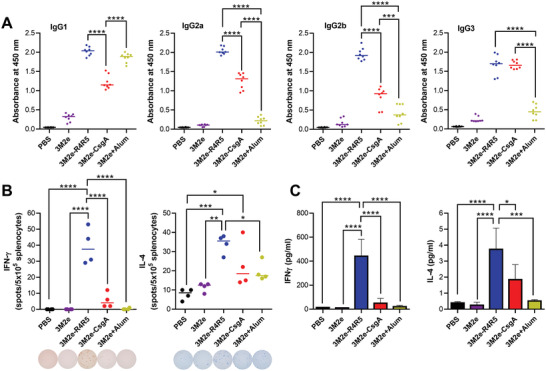
3M2e‐R4R5 nanofilaments induce a robust M2e‐specific cellular immune response. A) M2e‐specific IgG isotypes in mice sera following the 3rd immunization. B) IFN*γ* and IL‐4 ELISpot analysis of ex vivo splenocytes stimulated for 36 h with 2 µg of M2e peptide. C) ELISA analysis of IFN*γ* and IL‐4 secretion by splenocytes stimulated for 72 h with M2e peptide. Data represent mean ± S.E.M. (A) *n* = 8 per group. (B,C) *n* = 4 per group. (A–C) Statistical significance was obtained following one‐way ANOVA analysis with Tukey's multiple comparisons tests (*P < 0.05; **P < 0.01; ***P < 0.001; ****P < 0.0001).

Considering the high protection conferred by the 3M2e‐R4R5 platform compared to 3M2e‐CsgA nanofilaments, the polarization of the M2e‐specific immune response was analyzed to provide mechanistic insights into protection. In the context of vaccination against respiratory viruses, a balanced Th1/Th2 response is associated with tissue protection, whereas Th2 polarization could be linked to enhanced respiratory disease pathologies.^[^ ^42^ ^]^ Initially, to assess the polarization of the M2e‐specific immune response, the IgG isotypes present in the sera of mice 14 days after the third immunization were monitored. IgG isotyping is an indicator of T helper polarization with IgG1 being associated with a Th2 response and IgG2a/IgG2b/IgG3 being related to a Th1 polarization.^[^ ^43^ ^]^ Mice immunized with the 3M2e‐R4R5 vaccine showed the highest level of IgG1 (**Figure** **6**A), whereas the IgG1 from 3M2e‐immunized mice without Alum was barely detectable. Levels of IgG2a and IgG2b were significantly higher for mice immunized with 3M2e‐R4R5, compared to mice that received 3M2e‐CsgA and 3M2e ± Alum. This observation could potentially explain the 100% protection conferred by 3M2e‐R4R5 since IgG2a is the most efficient subclass for IgG‐mediated Fc effector functions in mice, which are necessary for M2e‐mediated control of IAV infection.^[^ ^44^ ^]^ Levels of IgG3 were similar between the 3M2e‐CsgA and the 3M2e‐R4R5 groups while being significantly higher compared to 3M2e ± Alum. As anticipated from the known Th2‐polarized immune response associated with Alum,^[^ ^45^ ^]^ mice immunized with 3M2e + Alum showed low levels of Ig2a, IgG2b, and IgG3. Next, the cellular immune response was analyzed by collecting spleens seven days after the last boost and by restimulating the isolated splenocytes with synthetic M2e peptide. The secretion of interferon‐gamma (IFN*γ*) and interleukin‐4 (IL‐4), which are respectively associated with a Th1 and Th2 responses, was assessed using ELISpot. Upon ex vivo restimulation with the M2e peptide, robust IFN*γ*, and IL‐4 responses were detected in splenocytes isolated from mice immunized with the 3M2e‐R4R5 formulation (Figure 6B). Splenocytes from mice immunized with 3M2e‐CsgA only showed a modest IL‐4 response while the IFN*γ* production was not significantly higher than PBS‐immunized mice. No significant IFN*γ* and IL‐4 responses were detected from mice that received 3M2e ± Alum. Furthermore, the cytokine levels in the supernatant of M2e‐stimulated splenocytes were measured by ELISA and the splenocytes of 3M2e‐R4R5 immunized mice showed the highest secretion of IFN*γ* and IL‐4 (Figure 6C). Intriguingly, the secretion of IL‐4 from splenocytes of mice inoculated with 3M2e‐CsgA was not significantly higher than the PBS‐immunized mice.

The robust cellular immune response directed toward M2e in 3M2e‐R4R5 immunized mice likely explains why these mice were completely protected against IAV infection. For instance, it was reported that CD4^+^ T cells are critical for protection conferred by the universal influenza vaccine in mice^[^ ^46^ ^]^ and that M2e‐specific IFN*γ* secretion could promote the recruitment of macrophages and natural killer cells, facilitating the elimination of infected cells.^[^ ^47^ ^]^ The higher magnitude of cellular and humoral immune responses observed in mice immunized with 3M2e‐R4R5, compared to 3M2e‐CsgA, is intriguing. This discrepancy could be related to the density of the M2e peptide on the nanofilament surface, as the absence of the first three repeating units of CsgA would lead to higher M2e valency, which could enhance B cell activation and proliferation following BCR cross‐linking^[^ ^48^ ^]^ and efficiently engage T cells.^[^ ^49^ ^]^ Besides, the 3M2e‐CsgA formulation tends to precipitate into large aggregates of fibrils (Figures S3 and S4, Supporting Information), which could potentially limit the processing of the nanovaccine by APCs. In sharp contrast, the 3M2e‐R4R5 nanofilaments remain well‐defined and soluble, and the vaccine formulation showed an high viscosity, which can promote retention time at the injection site.^[^ ^30b^ ^]^ Notwithstanding the molecular basis of the divergence between engineered CsgA‐based nanovaccines, these results highlight the high capacity of 3M2e‐R4R5 to induce a strong Th1/Th2 balanced immune response.

### 2.6 CsgA‐Based Nanovaccines Do Not Induce Adverse Overactivation of Inflammatory Response

**Figure 7:**
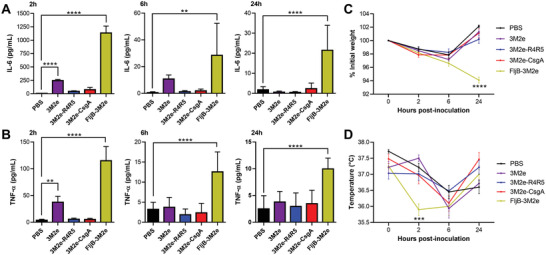
CsgA‐based nanofilaments do not induce apparent inflammation. A,B) Serum (A) IL‐6 and (B) TNF‐*α* levels 2, 6, and 24 h after IP inoculation with 20 µm of CsgA‐based nanofilaments, soluble 3M2e, or FljB‐3M2e. C) Percentage of initial weight and D) rectal temperature after IP inoculation. (A–D) *n* = 6 per group, data represent the mean ± S.D. and statistical significance was obtained following a two‐way ANOVA with Tukey's multiple comparison test (**P < 0.01; ***P < 0.001; ****P < 0.0001).

The usage of pathogen‐associated molecular patterns as vaccine adjuvants can be associated with an overstimulation of the immune system, which can lead to sustained systemic inflammation and off‐target toxicity.^[^ ^50^ ^]^ For instance, the bacterial protein flagellin (FljB), a TLR5 agonist that was evaluated in the clinics as an adjuvant, induces severe proinflammatory symptoms in immunized individuals associated with elevated inflammatory markers in the blood.^[^ ^51^ ^]^ Accordingly, the levels of the proinflammatory cytokines IL‐6 and tumor necrosis factor‐*α* (TNF‐*α*) in the sera were measured 2, 6, and 24 h after intraperitoneal (IP) inoculation, a route of administration promoting systemic exposure to antigenic materials.^[^ ^52^ ^]^ Following IP inoculation with CsgA‐based filaments, the levels of IL‐6 and TNF‐*α* in the sera were equivalent to the levels observed in mice inoculated with the PBS vehicle control (**Figure** **7**A,B). Mice inoculated with monomeric FljB‐3M2e, prepared as previously described,^[^ ^53^ ^]^ had significantly higher levels of both proinflammatory cytokines for all timepoints, which did not return to baseline 24 h after IP inoculation. Surprisingly, inoculation with the soluble 3M2e induced an increase of IL‐6 and TNF‐*α* in blood circulation 2 h after IP administration. Considering that the 3M2e soluble protein has a low molecular weight and can readily diffuse in the vasculature, the elevated levels of cytokines likely reflect systemic dispersion. By contrast, conjugation of 3M2e on engineered protein nanofilaments likely precludes the passive diffusion of the antigenic materials within the bloodstream upon inoculation. The rectal temperature and weight loss were also monitored. Mice that received FljB‐3M2e showed a decrease of body temperature 2 h postinoculation, and a significant weight loss was observed 24 h after inoculation (Figure 7C,D). By contrast, no weight loss and decrease in body temperature was observed in mice inoculated with 3M2e, 3M2e‐CsgA, and 3M2e‐R4R5 formulations. These results suggest that CsgA‐based nanovaccine formulations are safe and innocuous, although it remains critical to better investigate the full biosafety profile of CsgA‐based vaccines upon immunization. This safety analysis should include blood biochemistry, potential tissue damage at the injection site and anatomical pathology of major organs.

## 3 Conclusion

In this work, we reported a novel self‐assembling proteinaceous antigen‐delivery platform with intrinsic adjuvant properties that confers high protection to mice against IAV infection. Full‐length CsgA and a fragment comprising its R4R5 domain readily assembled into filaments that expose the antigen at their surface, while retaining their immunostimulatory properties. The 3M2e‐R4R5 nanofilaments stimulated robust antigen‐specific humoral and cellular immune responses, characterized by a balanced Th1/Th2 polarization, with limited adverse side effects. Interestingly, the M2e‐specific immune response induced by the 3M2e‐R4R5 formulation was more robust than the one induced by 3M2e‐CsgA. This discrepancy could be related to a better control over the self‐recognition and aggregation process and/or to the increase in viscosity of the vaccine formulation. Alternatively, the difference could be associated with the higher valency of antigens at the surface of R4R5 fibrils, which could promote B cell activation via BCR clustering and subsequent antigen presentation to T cells. Further studies looking at B cell activation and germinal center formation could potentially provide a clearer view of the mechanisms involved in this differential engagement of the adaptive immune system. Besides, it would be interesting to evaluate the binding affinity of the generated anti‐M2e antibodies and their capacity to bind to the M2 protein expressed on infected cells, since this could also explain the significant difference in survival between 3M2e‐CsgA and 3M2e‐R4R5 immunized mice. The R4R5‐based formulation led to key advantages for its usage as vaccine nanoscaffold; moderate solution viscosity, absence of large aggregates that precipitate, purification under native conditions allowing the delivery of conformational antigens, and no apparent systemic inflammation. As the shape, length, and size of antigen delivery nanoplatforms are known to modulate their immunogenicity,^[^ ^7a^ ^]^ it will be critical to further investigate how the suprastructural heterogenicity of the vaccine formulation affect the immune responses induced by CsgA nanostructures. Especially, a better understanding of the relationships between the length of CsgA filaments and their immunostimulatory properties would be particularly important before potential clinical translation. The potent in vivo immunostimulating properties of CsgA assemblies are likely associated with a combination of biological effects, including increased uptake by APCs, improved proteolytic stability of the grafted antigen, activation of immune cells through TLR2 and/or enhanced depot effect. In this view, it will be critical to delineate the specific contribution of TLR2 activation in the mechanisms of CsgA‐based nanovaccines using TLR2 knock‐out mice. Overall, the present study highlights for the first time the potential of engineered CsgA‐based nanostructures as safe, effective, and versatile immunostimulatory antigen delivery systems for usage in next‐generation vaccines to fight infectious diseases.

## 4 Experimental Section

### Protein Expression, Purification, and Self‐Assembly

The pET‐29a(+) plasmids containing the 3M2e‐CsgA, 3M2e‐R4R5, eGFP‐CsgA, eGFP‐R4R5, and 3M2e sequences were generated from GeneScript services. The N‐terminal secretion signal was removed from the CsgA sequence (Genbank accession number: WP_074524256.1). The tandem repeats of M2e were spaced from CsgA, or R4R5, by a flexible GGGSGGGS linker and a His‐tag (6×His) was inserted at the C‐terminus. Plasmids were transformed in *E. coli* NiCo21(DE3) and cells were grown to an optical density at 600 nm of 1 before inducing expression with 0.5 mm isopropyl‐thiogalactopyranoside for 1 h at 37°C. Cells were then harvested. For purification of 3M2e‐CsgA and 3M2e‐R4R5, cells were lysed with 8 m guanidium hydrochloride (Gdn‐HCl) followed by sonication and centrifugation to pellet large debris. Nickel resin Profinity IMAC was added to the cell lysate and incubated for 1 h while tumbling at RT. The lysate was passed through a column and the resin was washed extensively with ice‐cold PBS and then with ice‐cold PBS supplemented with 12.5 mm of imidazole. Proteins were eluted with 250 mm imidazole and desalted in PBS buffer with Sephadex G‐25 fine beads by centrifugation. For eGFP‐CsgA, eGFP‐R4R5, and 3M2e, cells were lysed with ice‐cold PBS supplemented with 1% (v/v) Triton X‐100, 5% (v/v) glycerol, 25 mm of sucrose, 1 mm of ethylenediaminetetraacetic acid (EDTA), complete‐mini protease inhibitor, and Pierce's nuclease and lysozyme followed by sonication (4 × 20 s) on ice and centrifugation at 4 °C to pellet the debris. Proteins were purified using nickel resin Profinity IMAC, as described above. Protein solutions were sterilized by filtration with a 0.2 µm PVDF filter. For immunization, endotoxins were removed using Pierce endotoxin‐removal spin columns. Removal of endotoxins in the solution was confirmed using the toxin sensor chromogenic LAL endotoxin assay, and endotoxin levels in all protein solutions used for immunological characterization were below the detection level of the LAL assay (0.03 EU mL^−1^ with U.S. standard Endotoxin). Protein concentrations were determined with BCA reagent, and the purity of protein solutions was evaluated by SDS‐PAGE. Freshly purified proteins were assembled at room temperature in PBS buffer without agitation for 24 h at a concentration of 600 µg mL^−1^. FljB‐3M2e was expressed and purified as recently described.^[^ ^53a^ ^]^

### Transmission Electron Microscopy

Protein solutions were sonicated and diluted to 60 µg mL^−1^ in H_2_O before being applied to a glow‐discharged 300 mesh copper carbon‐coated grid. Samples were dried and stained with 1.5% (w/v) uranyl formate for 1 min before air‐drying. Grids were imaged using an FEI Tecnai G2 Spirit Twin microscope at 120 kV and mounted with a Gatan Ultrascan 4000 4k × 4k CCD camera system.

### Circular Dichroism Spectroscopy

Protein solutions were diluted to 200 µg mL^−1^ in H_2_O and added to a 1 mm pathlength quartz cell for analysis with a Jasco J‐815 CD spectropolarimeter. Measurement was set every 0.5 nm between 260 and 190 nm with a scan rate of 10 nm min^−1^. The background was subtracted with the PBS buffer alone and the spectra were smoothed with the Savitsky–Golay algorithm at 11 points. Raw data were converted to mean residue ellipticity using the following formula

(1) $$\mathrm{MRE}\left({\deg\;\mathrm{cm}}^{2}\;{\mathrm{dmol}}^{-1}\right)=\frac{\mathrm{CD}\;\mathrm{signal}\;\left(\mathrm{mdeg}\right)\times 10^{5}}{\mathrm{pathlenght}\;\left(\mathrm{cm}\right)\times \mathrm{protein}\;\mathrm{concentration}\;\left(\mu M\right)\times \mathrm{number}\;\mathrm{of}\;\mathrm{residues}}$$

### Fluorescence Spectroscopy

Thioflavin T was added to the protein samples at a final concentration of 40 µm, and the fluorescence emission was measured between 450 and 550 nm with excitation at 440 nm in a QuantaMaster 40 spectrofluorometer. ANS was added to the protein samples at a final concentration of 450 µm, and the fluorescence emission was measured between 385 and 550 nm with excitation at 370 nm.

### Measurement of Solution Turbidity

Turbidity of protein solutions was measured between 600 and 400 nm in a 10 mm pathlength quartz cell with a UV‐1280 Shimadzu spectrophotometer.

### Measurement of Viscosity

Analysis of solution viscosity was performed with the SV‐10 viscosimeter at room temperature using 10 mL cells.

### Antigen Accessibility by ELISA

High binding 96‐well microplates were coated overnight with 1 µm of CsgA‐based assemblies, or 3M2e, in 50 mm sodium carbonate buffer pH 9.6 at 4 °C. Following washing with PBS + 0.05% Tween 20 (PBS‐Tween), wells were blocked for 1 h with 1% w/v of bovine serum albumin in PBS‐Tween at RT. Wells were washed with PBS‐Tween and incubated for 2 h at room temperature with twofold dilutions of anti‐influenza M2 14C2 monoclonal antibody starting at a 1:250 dilution in blocking buffer. Plates were washed with PBS‐Tween and incubated 1 h at room temperature with horseradish peroxidase (HRP)‐conjugated goat anti‐mouse IgG at a dilution of 1:20 000. Plates were extensively washed and 3,3′–5,5′‐tetramethylbenzidine (TMB) substrate was added for 15 min at room temperature. The reaction was stopped with 1 N sulfuric acid (H_2_SO_4_), and the optical density at 450 nm was measured. Data are presented as a function of dilution of primary antibody.

### TLR Activation

HEK‐Blue hTLR2‐TLR1 and HEK‐Blue mTLR5 (InvivoGen) were respectively cultured in DMEM supplemented with 4.5 g L^−1^ of glucose, 2 mm of l‐glutamine, 10% (v/v) of fetal bovine serum (FBS), 100 U mL^−1^ of penicillin–streptomycin and 100 µg mL^−1^ of Normocin at 37 °C under 5% CO_2_. At roughly 80% confluence, cells were seeded in 96‐well plates at a density of 50 000 cell per well in HEK‐Blue detection medium and incubated for 16 h with protein solutions at the indicated concentrations. The colorimetric reaction was evaluated by measuring the absorbance at 620 nm.

### Secretion IL‐1*β* by Macrophages

J774.A1 murine macrophages were cultured in DMEM supplemented with 10% (v/v) of FBS and 100 U per mL of penicillin–streptomycin at 37 °C under 5% CO_2_. Cells were seeded in 24‐well plates at a density of 100 000 cells per well and incubated for 16 h with CsgA‐based assemblies and respective controls. Supernatants were collected, and the amount of IL‐1*β* was determined by sandwich ELISA according to the manufacturer procedures.

### Cell Viability

For metabolic activity, J774.A1 cells were seeded in 96‐well plates at a density of 30 000 cells per well and incubated for 16 h with CsgA‐based assemblies. Resazurin (50 µm) was added and, after 4 h incubation, the absorbance at 570 nm was measured. Cell viability (in %) was calculated from the ratio of the fluorescence of the treated sample to the PBS control‐treated cells. For LIVE/DEAD assay, J774.A1 cells were seeded in 8‐chamber coverslips at a density of 100 000 cell per chamber and, the following day, cells were incubated for 16 h with CsgA‐based assemblies. Cell media was removed, and cells were stained with 4 µm of ethidium homodimer and 2 µm of calcein‐AM in PBS for 30 min at room temperature before imaging on a Nikon A1R confocal microscope. Control dead cells were obtained by a 10 min treatment with 70% methanol.

### Cellular Uptake

J774.A1 and DC2.4 cells were seeded in 24‐well plates at a density of 200 000 cells per well. After overnight incubation, eGFP or eGFP‐R4R5 nanofilaments were added to the cell media and cells were incubated at 37 °C for 3 h before extensive washing with PBS. Cells were analyzed in a BD Accuri flow cytometer with excitation at 488 nm and emission at 525 nm following quenching with 50% (v/v) of trypan blue 0.4% to remove membrane‐associated fluorescence. The FlowJo program was used to determine eGFP median fluorescence intensity. For confocal microscopy, J774.A1 macrophages and DC2.4 cells were seeded in 8‐chamber coverslips at a density of 50 000 cells per chamber. After overnight incubation, cells were incubated with eGFP or eGFP‐R4R5 filaments for 3 h before extensive washing with PBS. Cells were fixed with 4% formaldehyde for 10 min and stained for 30 min at room temperature with 0.5 µg mL^−1^ 4′,6‐diamidino‐2‐phenylindole dihydrochloride (DAPI) and 0.165 µm Texas Red‐X phalloidin. Cells were imaged using a Nikon A1R confocal microscope with a 60× oil immersion lens (405, 488, and 562 nm lasers excitation). Images were analyzed using the ImageJ software and are presented as Z‐stack.

### Activation of Dendritic Cells

DC2.4 cells were seeded in 24‐well plates at a density of 200 000 cells per well and treated for 24 h with CsgA‐based assemblies. Cells were washed with FACS buffer (PBS 2% FBS (v/v), 2 mm EDTA) and incubated for 30 min in Fc block (2.4G2 hybridoma supernatant). Cells were stained with anti‐mouse MHC‐II (M5/114.15.2) PE‐Cy5 monoclonal antibody and anti‐CD80 (16‐10A1) PE‐Cy7 monoclonal antibody at 1 µg mL^−1^ for 45 min, and then fixed for 10 min as previously described. Fluorescence was measured using a Beckman Coulter CytoFLEX cytometer and data were analyzed using FlowJo software.

### Mice Immunization

The animal protection committee of the Université du Québec à Montréal authorized all experiments with animals (0320‐R3‐924‐0321), in agreement with Canadian guidelines and regulations. Six‐to‐eight weeks old female BALB/c mice were placed under isoflurane anesthesia and immunized intramuscularly with 18 µg of 3M2e or equimolar doses of 3M2e‐CsgA (50 µg) or 3M2e‐R4R5 (30 µg) diluted in 100 µL of endotoxin‐free PBS, or 100 µL PBS:Alum (1:1) for the Alum‐supplemented formulation. Weights were monitored every day following immunization. Three immunizations were performed 14 days apart and sera were collected the day before to the immunization via the saphenous vein. In each group, four out of 12 mice were sacrificed 13 days after the second boost to collect blood by cardiac puncture for antibodies isotyping. The sera of the other eight mice were collected via the saphenous vein 13 days after the third immunization.

### Antibody Titers by ELISA

High‐binding ELISA plates were coated with 2 µg mL^−1^ of M2e synthetic peptide in 50 mm sodium carbonate buffer pH 9.6 at 4 °C. After blocking, plates were washed with PBS‐Tween and incubated for 2 h at RT with twofold dilutions of sera starting at a 1:65 dilution in blocking buffer. Plates were washed with PBS‐Tween and incubated for 1 h at room temperature with HRP‐conjugated goat antimouse IgG at a dilution of 1:20 000. After washing with PBS‐Tween, TMB substrate was added for 15 min at room temperature. The reaction was stopped with 2 N H_2_SO_4_ and the O.D. at 450 nm was measured. The O.D. at 450 nm was plotted against dilution of sera by means of a regression curve: (*y* = (*b* + *cx*)/(1 + *ax*)). The antibody titer was established as the highest sera dilution associated with an absorbance value twice that of the blank control (no primary antibody/no sera). For IgG isotypes, HRP‐conjugated goat antimouse antibodies were diluted 1:30 000. Data are represented as O.D. at 450 nm at a given dilution; IgG1:1:16 000; IgG2a:1:1000; IgG2b:1:1000; IgG3:1:65.

### ELISpot

Spleens were collected from immunized mice seven days after the third dose and splenocytes were extracted with a 70 µm cell strainer, and red blood cells were lysed using red blood cells lysis buffer. Mouse IFN*γ*/IL‐4 dual color ELISpot kit (ImmunoSpot CTL) was used to measure the production of both cytokines following ex vivo stimulation. ELISpot 96‐well plates were coated overnight with capture antibodies. Wells were washed with PBS and blocked with splenocytes media (RPMI‐1640 supplemented with 10% FBS, 2 mm l‐glutamine, 1 mm HEPES, 4.5 g L^−1^ of glucose, 1.5 g L^−1^ of sodium bicarbonate, 50 µm of 2‐mercaptoethanol, and 100 U mL^−1^ of penicillin–streptomycin) for 2 h at 37 °C. Splenocytes were seeded at a density of 500 000 cells per well in 96‐well ELISpot plates and stimulated with 10 µg mL^−1^ of M2e synthetic peptide for 36 h at 37 °C under 5% CO_2_. Spots were detected by alkaline phosphatase‐conjugated (IL‐4) or HRP‐conjugated (IFN*γ*) antibodies, and the number of spots was calculated with an ELISpot plate reader. Control splenocytes were stimulated with 10 µg mL^−1^ of the E2EP3 peptide, which is derived from the Chikungunya virus, or with a combination of 250 ng mL^−1^ of phorbol 12‐myristate 13‐acetate and 500 ng mL^−1^ of ionomycin.

### Secretion of IFN*γ* and IL‐4 from Isolated Splenocytes

Isolated splenocytes were seeded in 96‐well tissue culture treated plates and stimulated with 10 µg mL^−1^ of M2e peptide. After 72 h, the supernatants were collected to quantify cytokine production using sandwich ELISA for IFN*γ* and IL‐4.

### IAV Experimental Challenge

Fourteen days after the third immunization, mice were anesthetized with isoflurane and instilled with 5 × LD_50_ of influenza virus A/Puerto Rico/8/1934/H1N1. Weight and clinical scores were monitored twice every day (Table S1, Supporting Information). Mice that had lost more than 20% of their initial weight or showed clinical signs of severe symptoms were sacrificed by isoflurane inhalation followed by cervical dislocation.

### Evaluation of Proinflammatory Response

Six‐to‐eight weeks old female BALB/c mice were inoculated intraperitoneally with 20 µm of proteins in 50 µL of endotoxin‐free PBS. FljB‐3M2e chimeric protein was obtained as previously described.^[^ ^53a^ ^]^ Weight and rectal temperature were monitored at 2, 6, and 24 h postinoculation, and mice were sacrificed, after cardiac puncture under isoflurane anesthesia, by cervical dislocation. Levels of IL‐6 and TNF‐*α* in the sera were measured using sandwich ELISA kits.

### Statistical Analysis

Data were expressed as arithmetic means ± S.D., or as mean ± S.E.M., as stated in the corresponding figure legends. One‐way or two‐way analysis of variance (ANOVA) with Tukey's multiple‐comparison test, or log‐rank Mantel‐Cox test (> two groups) was used to compare unpaired values (GraphPad software), as stated in the corresponding figure legends. *P* values of <0.05 were considered significant; levels of significance are indicated on the graphs by asterisks with; *P < 0.05; **P < 0.01; ***P < 0.001; ****P < 0.0001.

## Conflict of Interest

The authors disclose a patent pending related to the present study entitled “CsgA‐derived nanostructures and uses thereof for antigen delivery” USPTO, Application No. 63/374770.

## Supporting information

Supporting Information

## Data Availability

The data that support the findings of this study are available from the corresponding author upon reasonable request.
