# Supplementary material for: Engineered Curli Nanofilaments as a Self‐Adjuvanted Antigen Delivery Platform
Source: Adv Healthc Mater. 2023 Apr 29;12(21):2300224. doi: 10.1002/adhm.202300224 (PMC11468023; doi:10.1002/adhm.202300224)
Supplement: Supplementary file 1 — Supporting Information [file ADHM-12-2300224-s001.pdf]

ADVANCED HEALTHCARE MATERIALS

Supporting Information

for *Adv. Healthcare Mater.*, DOI 10.1002/adhm.202300224

Engineered Curli Nanofilaments as a Self-Adjuvanted Antigen Delivery Platform

Félix Lamontagne, Dominic Arpin, Mélanie Côté-Cyr, Vinay Khatri, Philippe St-Louis, Laurie Gauthier, Denis Archambault and Steve Bourgault**

Supporting Information

Engineered Curli Nanofilaments as a Self-Adjuvanted Antigen Delivery Platform

Félix Lamontagne, Dominic Arpin, Mélanie Côté-Cyr, Vinay Khatri, Philippe St-Louis, Laurie Gauthier, Denis Archambault*, Steve Bourgault*

*Corresponding authors:
Denis Archambault, C.P. 8888, Succursale Centre-Ville, Montreal, H3C 3P8, Canada; e-mail address: archambault.denis@uqam.ca

Steve Bourgault, C.P. 8888, Succursale Centre-Ville, Montreal, H3C 3P8, Canada; e-mail address: bourgault.steve@uqam.ca

Table of contents

Figure S1. Flow chart of the preparation of CsgA-based nanovaccines………………… 3

Figure S1. SDS-PAGE analysis of purified proteins……………………………………. 4

Figure S2. Representative TEM images of 3M2e-CsgA and 3M2e-R4R5 assemblies… 5

Figure S3. Representative AFM images of 3M2e-CsgA and 3M2e-R4R5 assemblies… 6

Figure S4. Length distribution of 3M2e-R4R5 nanofilaments………………………….. 7

Figure S5. Assessment of solution viscosity by tube inversion…………………………. 8

Figure S6. SDS-PAGE analysis of purified eGFP-R4R5……………………………….. 9

Figure S7. Representative TEM image of assembled eGFP-R4R5……………………… 10

Figure S8. eGFP-R4R5 assemblies activate TLR2-TLR1………………………………. 11

Figure S9. Cellular uptake by DC2.4 dendritic cells…………………………………… 12

Figure S10. Cellular uptake by J774.A1 macrophages………………………………….. 13

Figure S11. Flow cytometry analysis of cell uptake by DC2.4 cells……………………. 14

Figure S12. Flow cytometry analysis of cell uptake by J774.A1 macrophages…………. 15

Table S1. Scale for clinical symptoms after IAV infection…………………………….... 16

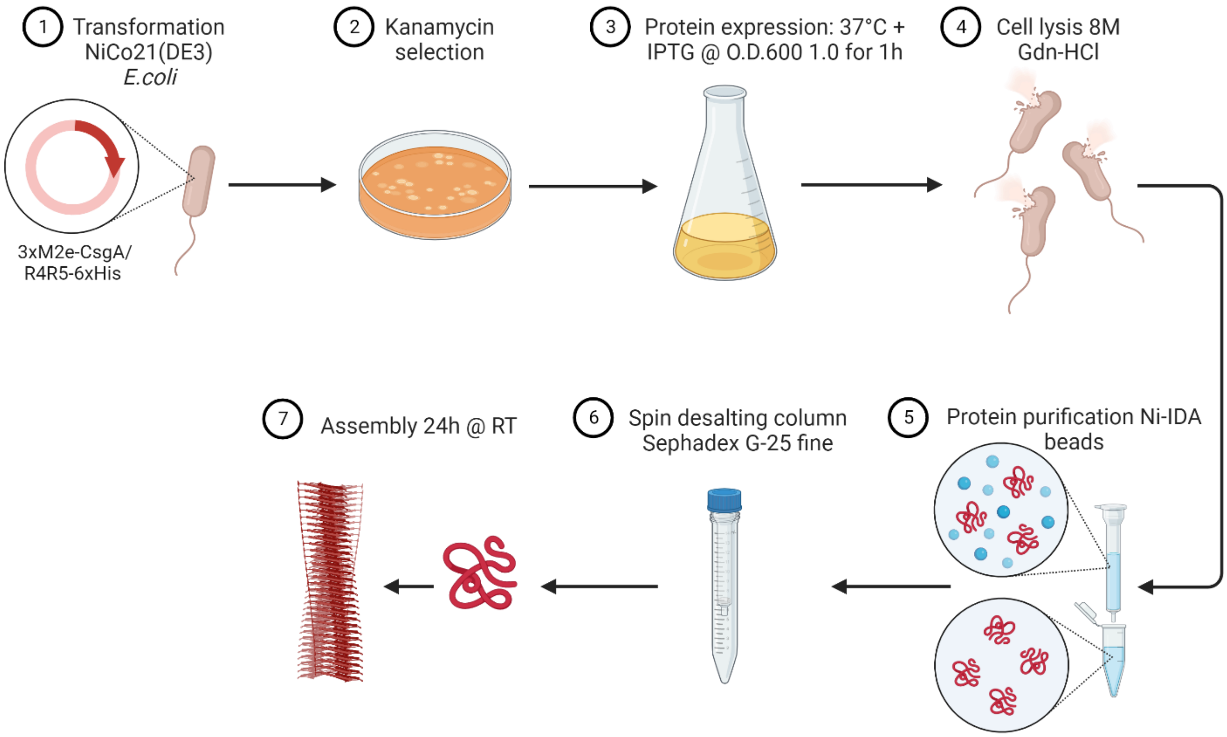

Figure S1. Flow chart summarizing the preparation of CsgA-based nanovaccines.

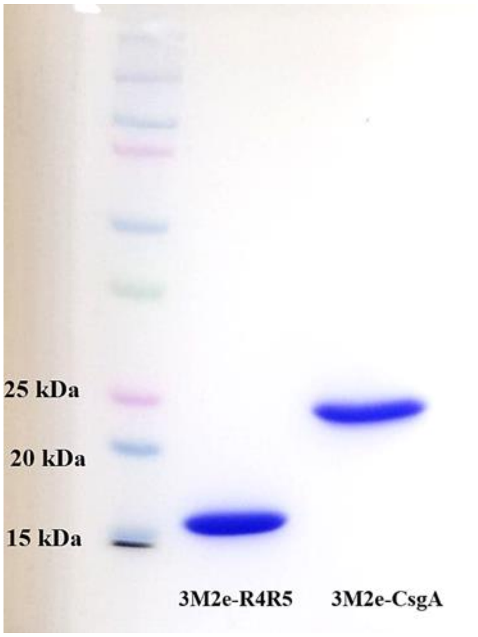

Figure S2. Coomassie Blue stained SDS-PAGE analysis of purified 3M2e-R4R5 and 3M2e-CsgA proteins.

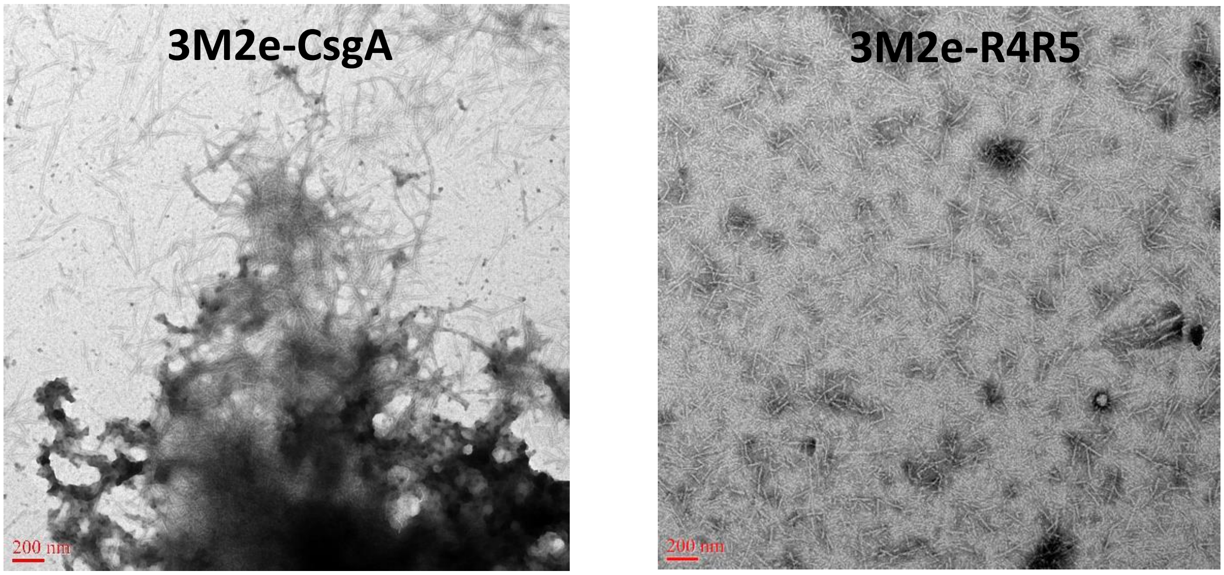

Figure S3. Representative TEM negatively stained images of aggregates formed by the assembly of 3M2e-CsgA and 3M2e-R4R5. Proteins were assembled in PBS for 24 h at room temperature at a concentration of 600 µg/ml under fully quiescent conditions.

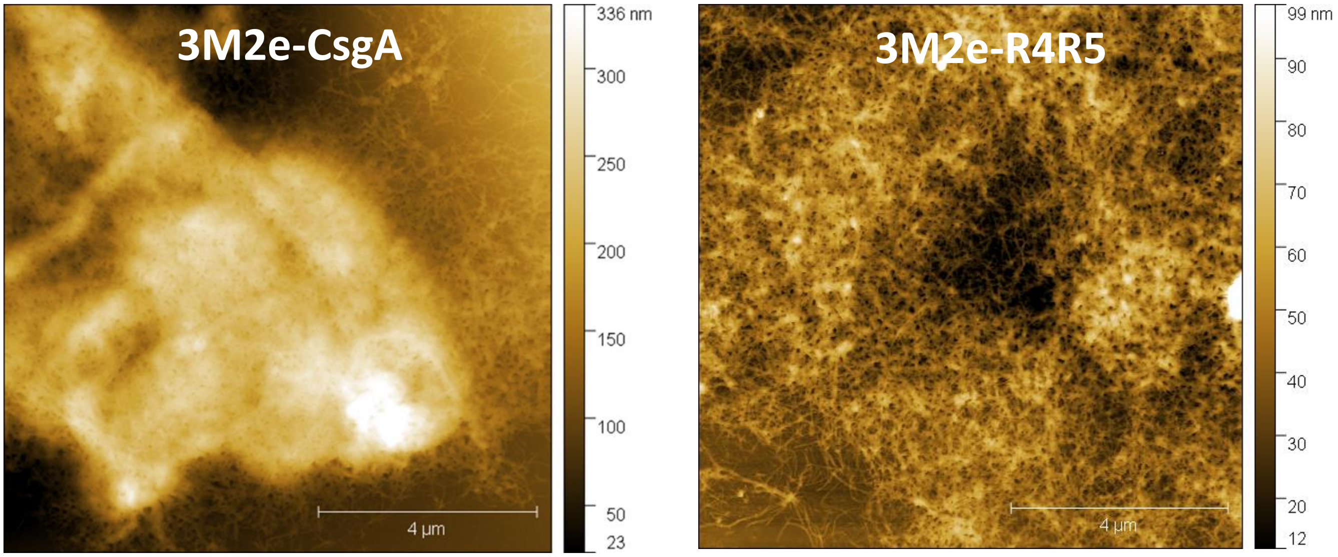

Figure S4. Representative AFM images of aggregates formed by the assembly of 3M2e-CsgA and 3M2e-R4R5. Proteins were assembled in PBS for 24 h at room temperature at a concentration of 600 µg/ml under fully quiescent conditions.

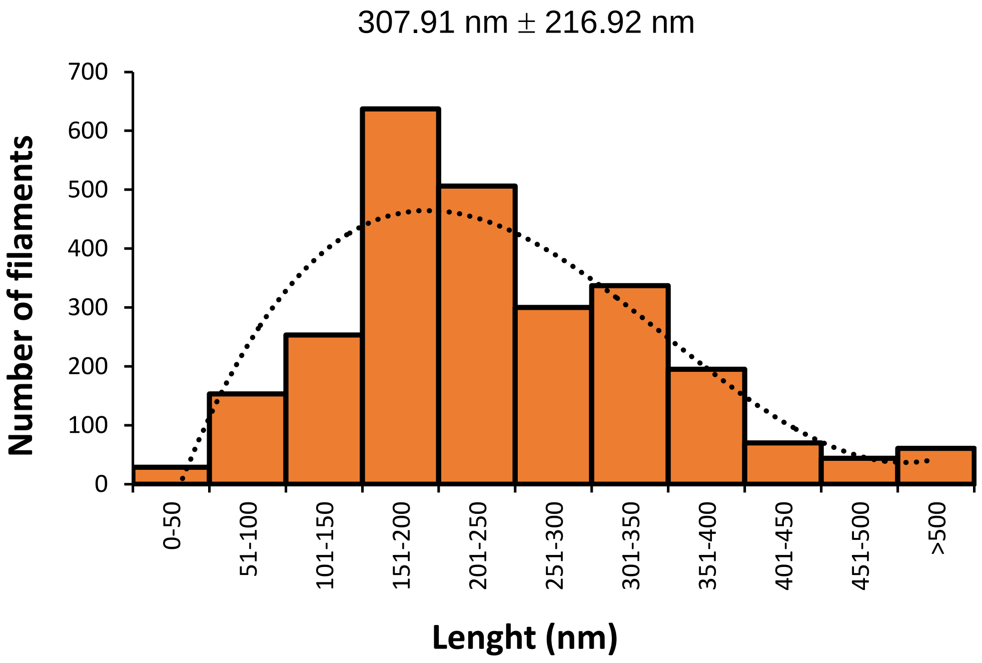

Figure S5. Length distribution of 3M2e-R4R5 nanofilaments extracted from the analysis of transmission electron microscopy images.

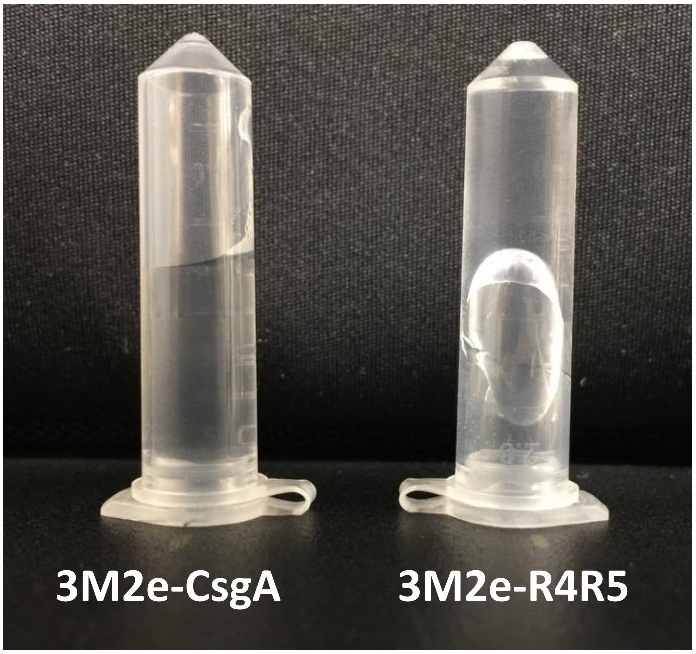

Figure S6. Assessment of solution viscosity by tube inversion of assembled 3M2e-CsgA and 3M2e-R4R5. Proteins were assembled in sterile PBS for 24 h at room temperature at a concentration of 600 µg/ml under fully quiescent conditions.

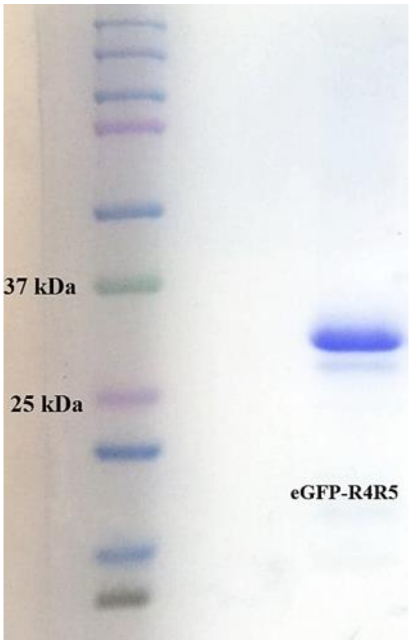

Figure S7. Coomassie Blue stained SDS-PAGE analysis of purified eGFP-R4R5.

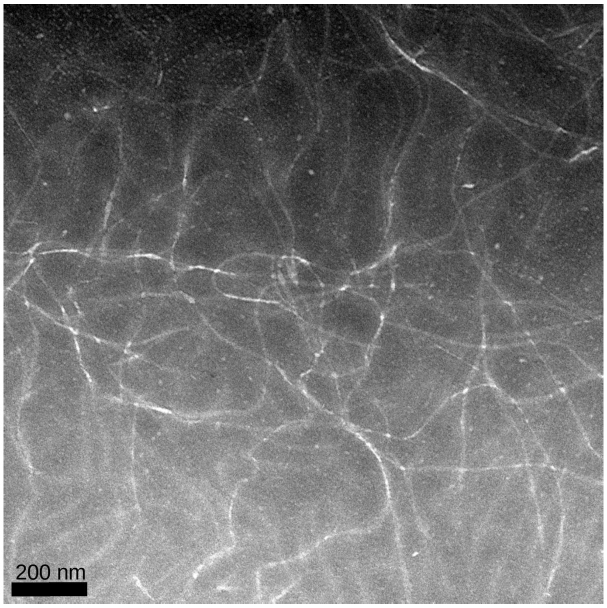

Figure S8. Representative TEM image of assembled eGFP-R4R5. eGFP-R4R5 chimeric protein was assembled for 24 h in PBS at a concentration of 600 µg/ml under quiescent conditions and room temperature.

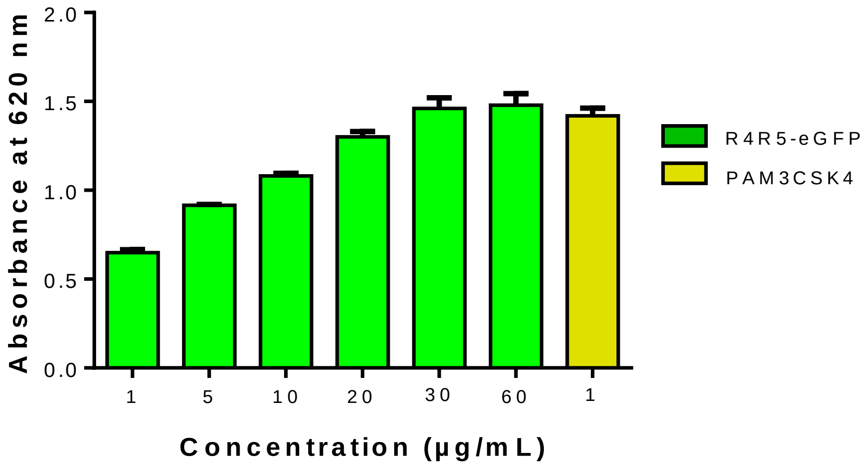

Figure S9. eGFP-R4R5 assemblies activate TLR2-TLR1. HEK-Blue cells expressing the heterodimer TLR2-TLR1 were exposed to nanofilaments for 16 h and activation was measured using SEAP reporter.

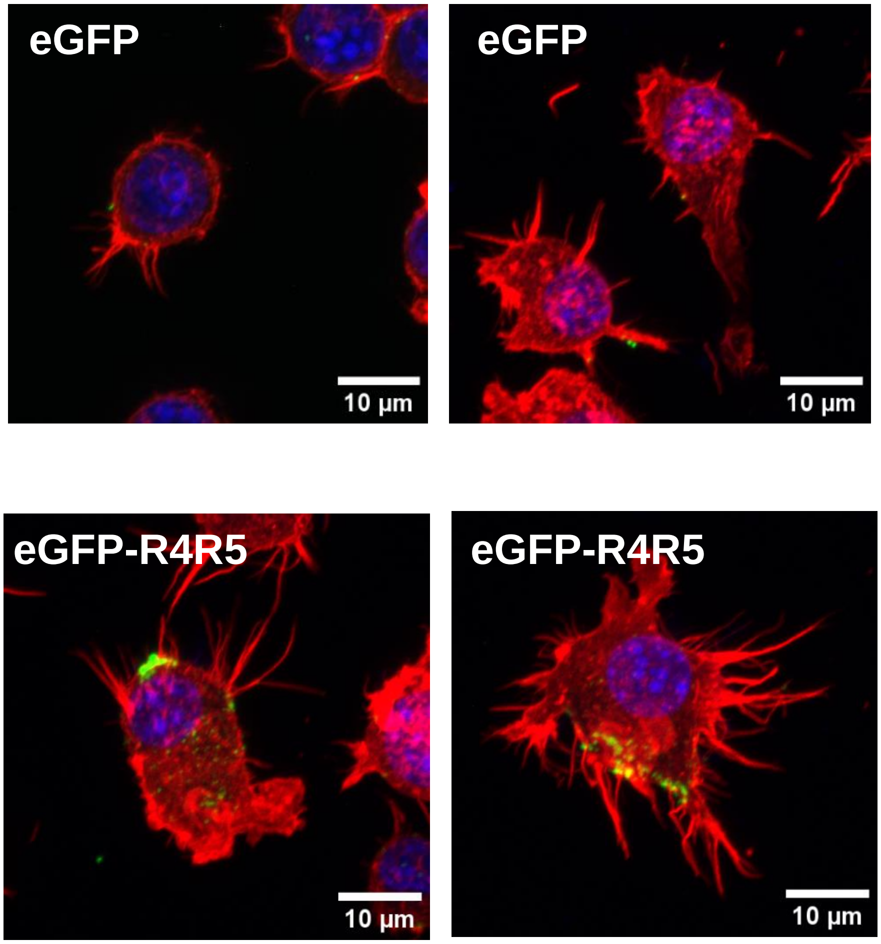

Figure S10. Cellular uptake by DC2.4 dendritic cells. DC.2.4. cells were treated 30 µg/mL for 3 h with eGFP or eGFP-R4R5 nanofilaments followed by extensive washing before imaging by confocal fluorescence microscopy.

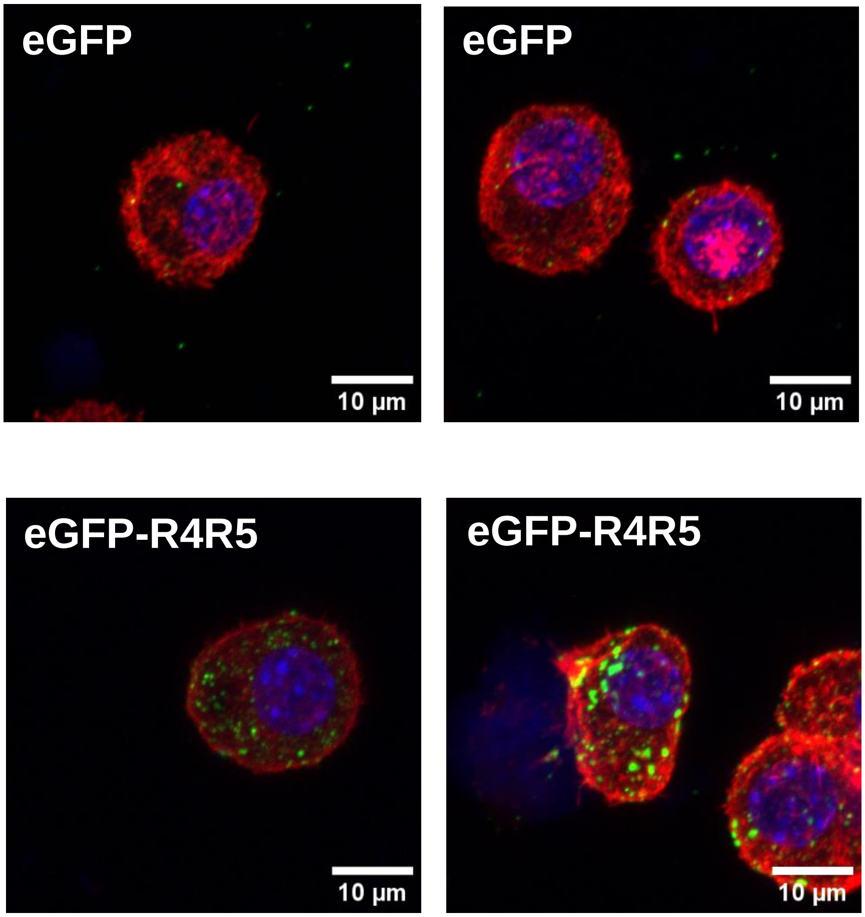

Figure S11. Cellular uptake by J774.A1 macrophages. J774.A1 cells were treated 30 µg/mL for 3 h with eGFP or eGFP-R4R5 nanofilaments followed by extensive washing before imaging by confocal fluorescence microscopy.

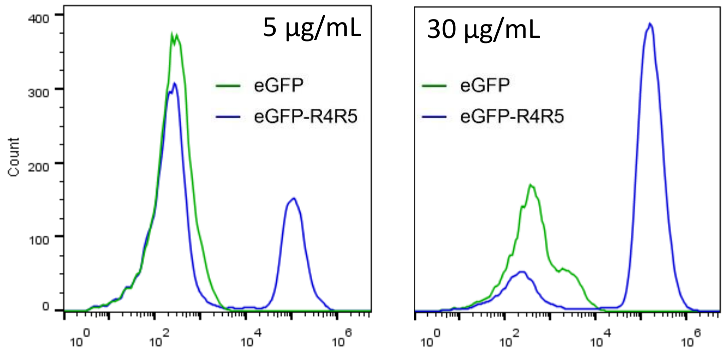

Figure S12. Flow cytometry analysis of cell uptake by DC2.4 cells. Representative flow cytometry histograms showing the internalization of 5 and 30 µg/mL of eGFP and eGFP-R4R5 nanofilaments after 3 h incubation at 37°C.

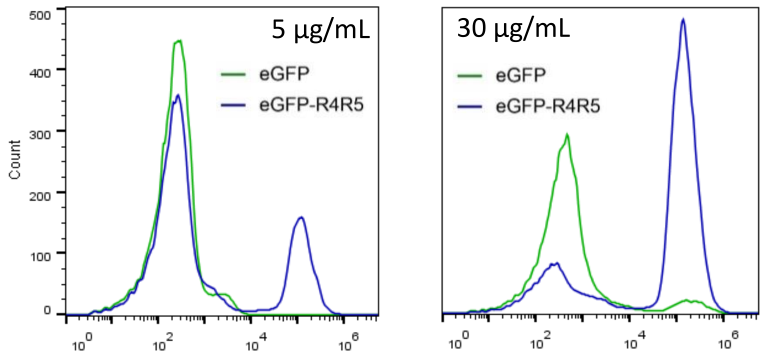

Figure S13. Flow cytometry analysis of cell uptake by J774.A1 macrophages. Representative flow cytometry histograms showing the internalization of 5 and 30 µg/mL of eGFP and eGFP-R4R5 nanofilaments after 3 h incubation at 37°C.

Table S1. Scale for clinical symptoms after IAV infection.

**Intensity**	**Temperature (°C)**	**Fur**	**Posture**	**Eyes**	**Ears**	**Response to stimuli**	**Activity**	**Feces**	**Dehydration (Pinch on the skin)**
**0 (Absent)**	> 36	Smooth and even	Normal	Open	Normal	Normal	Normal	Normal	Normal
**1 (Light)**	35-36	Fur loss	Slightly hunched back	Half-closed	Bent	Calm, curious	Reduced	Soft	Skin rapidly recovers
**2 (Moderate)**	32-35					Delay in response	Immobile, reactive	Sticky	Skin slowly recovers
**3 (Severe)**	< 32	Ruffled fur	Hunched back	Closed	Laid down	Inactive	Lethargic	Liquid	No recovery
